# Supplementary material for: Alarming antibody evasion properties of rising SARS-CoV-2 BQ and XBB subvariants
Source: Cell. 2023 Jan 19;186(2):279–286.e8. doi: 10.1016/j.cell.2022.12.018 (PMC9747694; doi:10.1016/j.cell.2022.12.018)
Supplement: Table S1 Demographics of the clinical cohorts, related to Figure 2 [file mmc1.pdf]

Cell, Volume *186*

Supplemental information

Alarming antibody evasion properties of rising SARS-CoV-2 BQ and XBB subvariants

Qian Wang, Sho Iketani, Zhiteng Li, Liyuan Liu, Yicheng Guo, Yiming Huang, Anthony D. Bowen, Michael Liu, Maple Wang, Jian Yu, Riccardo Valdez, Adam S. Lauring, Zizhang Sheng, Harris H. Wang, Aubree Gordon, Lihong Liu, and David D. Ho

Table S1

Sample ID	Vaccine type and infected strain	Days post-vaccination or *infection	Documented COVID-19	Age	Gender
3 shots WT					
Q1	mRNA-1273/mRNA-1273/mRNA-1273	29	No	66	Female
Q2	BNT162b2/BNT162b2/BNT162b2	30	No	68	Male
Q3	BNT162b2/BNT162b2/BNT162b2	14	No	64	Female
Q4	BNT162b2/BNT162b2/BNT162b2	34	No	55	Male
Q5	BNT162b2/BNT162b2/BNT162b2	34	No	45	Male
Q6	BNT162b2/BNT162b2/BNT162b2	15	No	50	Female
Q7	BNT162b2/BNT162b2/BNT162b2	15	No	48	Female
Q8	BNT162b2/BNT162b2/BNT162b2	29	No	71	Male
Q9	BNT162b2/BNT162b2/BNT162b2	90	No	59	Male
Q10	BNT162b2/BNT162b2/BNT162b2	33	No	45	Male
Q11	BNT162b2/BNT162b2/BNT162b2	87	No	66	Female
Q12	BNT162b2/BNT162b2/BNT162b2	84	No	26	Male
Q13	mRNA-1273/mRNA-1273/mRNA-1273	23	No	28	Female
Q15	BNT162b2/BNT162b2/mRNA-1273	32	No	39	Male
4 shots WT					
UM-65	BNT162b2/BNT162b2/BNT162b2/BNT162b2	24	No	52	Female
UM-66	BNT162b2/BNT162b2/BNT162b2/BNT162b2	20	No	57	Female
UM-67	BNT162b2/BNT162b2/BNT162b2/BNT162b2	20	No	61	Female
UM-68	mRNA-1273/mRNA-1273/mRNA-1273/mRNA-1273	22	No	48	Female
UM-69	BNT162b2/BNT162b2/BNT162b2/BNT162b2	23	No	50	Female
UM-70	BNT162b2/BNT162b2/BNT162b2/BNT162b2	22	No	50	Female
UM-71	BNT162b2/BNT162b2/BNT162b2/BNT162b2	20	No	58	Female
UM-72	BNT162b2/BNT162b2/BNT162b2/BNT162b2	26	No	56	Female
UM-73	BNT162b2/BNT162b2/BNT162b2/BNT162b2	29	No	63	Female
UM-74	BNT162b2/BNT162b2/BNT162b2/BNT162b2	25	No	58	Female
UM-75	BNT162b2/BNT162b2/BNT162b2/BNT162b2	21	No	62	Male
UM-76	BNT162b2/BNT162b2/BNT162b2/BNT162b2	26	No	54	Female
UM-77	BNT162b2/BNT162b2/BNT162b2/BNT162b2	23	No	53	Male
UM-78	BNT162b2/BNT162b2/BNT162b2/BNT162b2	21	No	55	Female
UM-79	BNT162b2/BNT162b2/BNT162b2/BNT162b2	23	No	59	Female
UM-80	BNT162b2/BNT162b2/BNT162b2/BNT162b2	21	No	49	Female
UM-81	BNT162b2/BNT162b2/BNT162b2/BNT162b2	27	No	57	Female
UM-82	BNT162b2/BNT162b2/BNT162b2/BNT162b2	27	No	55	Female
Q97	BNT162b2/BNT162b2/BNT162b2/BNT162b2	36	No	53	Female
3 shots WT + bivalent					
UM-36	BNT162b2/BNT162b2/BNT162b2/Moderna Bivalent	24	No	38	Female
UM-37	BNT162b2/BNT162b2/BNT162b2/Moderna Bivalent	27	No	42	Female
UM-39	mRNA-1273//mRNA-1273/mRNA-1273/Moderna Bivalent	24	No	36	Male
UM-40	BNT162b2/BNT162b2/BNT162b2/Pfizer Bivalent	25	No	37	Female
UM-41	BNT162b2/BNT162b2/BNT162b2/Pfizer Bivalent	24	No	36	Male
UM-43	BNT162b2/BNT162b2/BNT162b2/Pfizer Bivalent	25	No	49	Female
UM-44	BNT162b2/BNT162b2/BNT162b2/Moderna Bivalent	25	No	37	Female
UM-47	BNT162b2/BNT162b2/BNT162b2/Pfizer Bivalent	26	No	45	Male
UM-48	BNT162b2/BNT162b2/mRNA-1273/Moderna Bivalent	26	No	43	Female
UM-51	mRNA-1273/mRNA-1273/mRNA-1273/Moderna Bivalent	29	No	32	Female
UM-52	BNT162b2/BNT162b2/BNT162b2/Pfizer Bivalent	23	No	43	Female
UM-53	BNT162b2/BNT162b2/BNT162b2/Pfizer Bivalent	26	No	43	Female
UM-54	BNT162b2/BNT162b2/mRNA-1273/Moderna Bivalent	29	No	38	Female
UM-55	BNT162b2/BNT162b2/BNT162b2/Moderna Bivalent	28	No	38	Female
UM-56	BNT162b2/BNT162b2/mRNA-1273/Moderna Bivalent	27	No	36	Female
UM-60	BNT162b2/BNT162b2/BNT162b2/Moderna Bivalent	30	No	24	Female
Q101	mRNA-1273/mRNA-1273/mRNA-1273/Moderna Bivalent	30	No	32	Female
Q102	BNT162b2/BNT162b2/mRNA-1273/Moderna Bivalent	23	No	39	Male
Q103	BNT162b2/BNT162b2/BNT162b2/Pfizer Bivalent	30	No	26	Female
Q104	mRNA-1273/mRNA-1273/mRNA-1273/Pfizer Bivalent	30	No	27	Female
Q105	BNT162b2/BNT162b2/BNT162b2/Pfizer Bivalent	23	No	23	Male
BA.2 breakthrough					
Q35	BNT162b2/BNT162b2/BA.2	*14	Yes	50	Female
Q36	BNT162b2/BNT162b2/BNT162b2/Ad26.COV2.S/BA.2	*22	Yes	69	Male
Q49	BNT162b2/BNT162b2/mRNA-1273/BA.2	*16	Yes	32	Male
Q50	mRNA-1273/mRNA-1273/mRNA-1273/BA.2	*14	Yes	34	Male
Q51	BNT162b2/BNT162b2/mRNA-1273/BA.2	*19	Yes	33	Female
Q52	BNT162b2/BNT162b2/mRNA-1273/BA.2	*18	Yes	29	Female
Q98	BNT162b2/BNT162b2/BA.2	*122	Yes	22	Male
Q99	mRNA-1273/mRNA-1273/BA.2	*164	Yes	30	Female
Q100	BNT162b2/BNT162b2/BA.2	*94	Yes	30	Female
A7	BNT162b2/BNT162b2/mRNA-1273/BA.2	*30	Yes	59	Female
A9	BNT162b2/BNT162b2/BNT162b2/BA.2	*29	Yes	39	Female
A11	BNT162b2/BNT162b2/BNT162b2/BA.2	*18	Yes	45	Female
A12	BNT162b2/BNT162b2/BNT162b2/BNT162b2/BA.2	*31	Yes	59	Female
A13	BNT162b2/BNT162b2/BNT162b2/BNT162b2/BA.2	*25	Yes	39	Male
BA.4/5 breakthrough					
Q71	mRNA-1273/mRNA-1273/BNT162b2/BA.5.2.1	*29	Yes	29	Female
Q77	BNT162b2/BNT162b2/BNT162b2/BA.5	*22	Yes	61	Female
Q79	mRNA-1273/mRNA-1273/mRNA-1273/BA.5	*15	Yes	28	Female
Q80	mRNA-1273/mRNA-1273/mRNA-1273/BA.5	*21	Yes	24	Female
Q81	BNT162b2/BNT162b2/BNT162b2/BA.5	*75	Yes	35	Female
Q82	BNT162b2/BNT162b2/mRNA-1273/BA.5	*63	Yes	46	Female
Q83	BNT162b2/BNT162b2/BNT162b2/BA.5	*28	Yes	55	Male
Q84	BNT162b2/BNT162b2/BNT162b2/BA.5	*17	Yes	57	Female
UM-85	BNT162b2/BNT162b2/BNT162b2/BA.5	*29	Yes	44	Female
UM-86	BNT162b2/BNT162b2/mRNA-1273/BA.5	*29	Yes	36	Female
UM-87	BNT162b2/BNT162b2/BNT162b2/BNT162b2/BA.5	*31	Yes	54	Female
UM-88	BNT162b2/BNT162b2/BNT162b2/BNT162b2/BA.5	*28	Yes	69	Male
UM-89	BNT162b2/BNT162b2/BNT162b2/BNT162b2/BA.5	*42	Yes	44	Male
UM-90	BNT162b2/BNT162b2/BNT162b2/BNT162b2/BA.5	*28	Yes	41	Female
UM-91	BNT162b2/BNT162b2/BNT162b2/BNT162b2/BA.5	*28	Yes	44	Female
UM-92	BNT162b2/BNT162b2/BNT162b2/BNT162b2/BA.5	*31	Yes	29	Female
UM-93	BNT162b2/BNT162b2/BNT162b2/BNT162b2/BA.5	*29	Yes	48	Female
UM-94	BNT162b2/BNT162b2/BNT162b2/BNT162b2/BA.5	*29	Yes	49	Female
UM-95	BNT162b2/BNT162b2/mRNA-1273/BNT162b2/BA.5	*28	Yes	37	Female
UM-96	BNT162b2/BNT162b2/BNT162b2/BNT162b2/BA.5	*33	Yes	58	Female
